# COVID-19-associated PTSD in the elderly—lessons learned for the next global pandemic

**DOI:** 10.1186/s43045-021-00119-3

**Published:** 2021-06-30

**Authors:** Ashish Sarangi, Sana Javed, Kumar Karki, Akshita Kaushal

**Affiliations:** 1 Texas Tech University Health Sciences Center, Lubbock, TX USA; 2 Nishtar Medical University, Multan, Pakistan; 3 National Medical College, Birgunj, Nepal; 4 Dr. Rajender Prasad Government Medical College and Hospital, Kangra, Himachal Pradesh India

**Keywords:** COVID-19, Coronavirus infection, SARSCoV2 infection, PTSD, Post-traumatic stress disorder

## Abstract

**Background:**

When COVID-19 was declared a global pandemic in March 2020, almost all countries implemented strict lockdowns and home quarantine orders in order to prevent spread of the virus. These implementations have severely affected the mental health of people all around the world especially the elderly, who are already physically and mentally fragile. There has been an escalation in the prevalence of depression, suicide, anxiety, substance abuse, domestic abuse, and post-traumatic stress disorder (PTSD).

**Main body:**

The aim of our review was to highlight PTSD in the elderly population who has recovered from COVID-19 infection and come up with some recommendations for the future. A thorough literature review was conducted focusing on the impact of COVID-19 on development and progression of PTSD during the pandemic.

**Conclusion:**

Increased allocation of resources by various government and private stakeholders is necessary to prepare for the long-term implications on mental health from the current and future pandemics.

## Background

The coronavirus disease (COVID-19) global pandemic has affected people’s lives in several ways. In addition to the directly apparent physical consequences of the disease, COVID-19 is continuing to affect people’s lives in socio-economic as well as psychological aspects [1, 2]. The risk of anxiety, insomnia, depressive disorders, and other mental abnormalities has increased due to bereavement, isolation, loss of income, and fear, and there has been an increase in alcohol and drug use [3, 4]. According to a US internet-based survey results published by Centers for Disease Control and Prevention (CDC) in August 2020 including more than 5000 adults, 40.9% of adults were found to have one or more COVID-19 pandemic-related adverse mental or behavioral health issues [5]. About 30.9% of adults demonstrated features of anxiety or depression, 13.3% had used substances for coping, and 10.7% seriously considered suicide in the prior days [5]. Post-traumatic stress disorder (PTSD), a psychiatric illness that occurs after experiencing or witnessing a terrifying incident includes symptoms like re-experiencing of the event as flashbacks and nightmares, avoidance of situations that cause reminiscence of the traumatic event, changes in mood (sadness, anger, and horror), and hyperarousal [6].

In this COVID-19 pandemic, people have been subjected to several traumatic stressors because of serious COVID-19 illness and potential death experienced by patients, witnessed suffering and death of patients by the family members, loss of a family member or a friend, and extreme exposure to aversive details (e.g., in journalists, medical personnel) [5]. Only a few studies have been performed to evaluate post-traumatic stress symptoms in the general population, though [5]. A study that had addressed the prevalence of PTSD among COVID recovered patients found that nearly 20% of these patients had symptoms of post-traumatic stress 1 month following hospitalization [7]. However, this study does not have a good representation of older adults above 65 years, who are the most vulnerable and the hardest-hit age group [7, 8]. Current literature on PTSD primarily focuses on stressors like physical assault, sexual violence, war, accident, kidnapping, and being threatened with a weapon, as the causative triggers of PTSD [9]. There is a minimum focus on infectious diseases as the trigger of PTSD. When infectious etiology is something like COVID-19 that can inflict several traumatic stressors on a significant portion of the world population, we believe that that has to be considered seriously.

The ongoing COVID-19 pandemic is likely not going to be the last one in history. We need to learn as much as possible from this pandemic to tackle the several problems associated with such a pandemic in a better way, should another such event occur in the future. Given the enormous impact of COVID-19 on the vulnerable elderly population, it is crucial to explore its effects on all aspects of their mental health, including post-traumatic stress-like symptoms. Therefore, we aim to review and discuss the correlation between COVID-19 and PTSD in the elderly population recovered from this infection.

## Methods

### Literature search

Two independent reviewers (AS, SJ) searched for articles on PubMed, Scopus, and Google Scholar. No time filter was applied during the database search. The key search terms “COVID-19” OR CORONAVIRUS INFECTION OR SARSCoV2 INFECTION AND “PTSD” OR POST TRAUMATIC STRESS DISORDER were used either alone or in combination to search for relevant articles. We applied filters for age 65+.

### Eligibility criteria

We included articles that have discussed post-traumatic stress disorder (PTSD) associated with COVID-19 in the geriatric population (65+ years) who are COVID-19 survivors. We looked for case reports, case series, observational studies, randomized controlled trials, and clinical trials. Those articles that have included people below 65 years were excluded. Letters and commentaries were excluded too.

### Data search and extraction

**Fig. 1:**
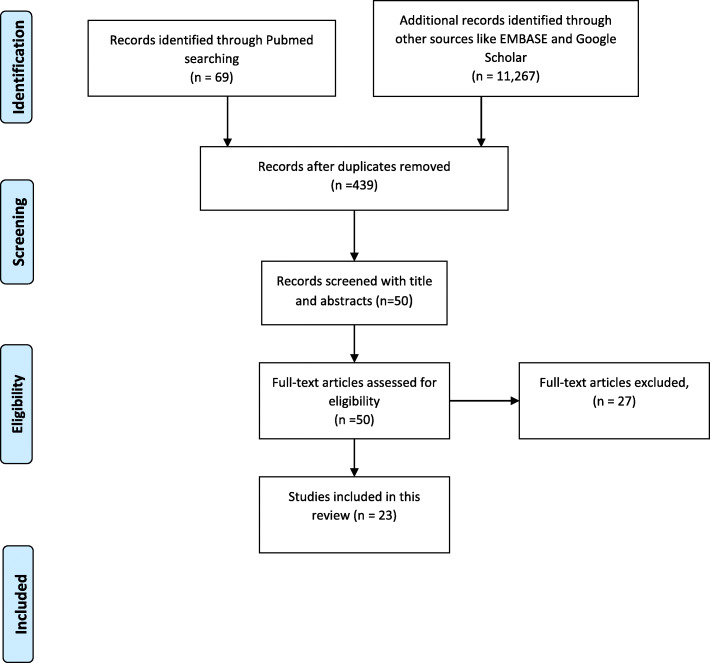
PRISMA flow diagram

The initial literature search on PubMed, Scopus, and Google Scholar identified a total of 11,336 articles. After removing duplicates and irrelevant articles by going through titles and abstracts, 50 articles were identified as potentially eligible. We read the full text of those 50 articles to find the studies that fit our eligibility criteria (Table 1). A total of 23 were included in our narrative review (Fig. 1).

**Table 1:** Table summarizing articles included as potentially eligible for review

Keywords	PubMed	Scopus	Google Scholar/EMBASE
“COVID-19” OR CORONAVIRUS INFECTION OR SARSCoV2 INFECTION AND "PTSD" OR POST	69	68	8940 + 2259

## Main text

In the past few years, there have been accounts of emerging but somehow contained deadly viral epidemics in different parts of the world, including Ebola Virus disease, Nipah Virus infection, or Zika Virus disease. Coronavirus disease has successfully become a global pandemic affecting millions of people worldwide. This pandemic has adversely affected every aspect of people’s lives ranging from economic, social, and physical to mental health. A significant proportion of people who survived the coronavirus disease face various respiratory and psychopathological sequelae [10]. The aim of this review is to systematically look at the existing literature regarding PTSD among the elderly to be armed with knowledge if any such global pandemic is to hit us further. The major limitation to the current literature is the method of obtaining results which is usually online and limits access to the people who are not avid technology users, thus leading to a general underrepresentation of people in the elder age group [11]. Even with this limitation, PTSD has been widely reported in the elderly worldwide.

A study from Spain has reported 6.81% of people from the elder age group suffering from PTSD during the COVID-19 pandemic [11]. Another study from France has reported a 9.9% incidence of PTSD in the elderly age group surviving the coronavirus disease [12]. 26.9% of older adults surviving COVID-19 had PTSD symptoms in a study from China with intrusion symptoms in 30.8%, avoidance symptoms in 30.8%, and hyperarousal symptoms in 30.8% [13]. In a study from Ireland, 1.6% of elderly study subjects had COVID-19-related PTSD [14]. Either factually or due to the limitation mentioned above, a very consistent finding in the current literature is the resilience to PTSD or other forms of psychiatric distress found in the older age group compared to the younger ones [11–20]. Most of studies link being female to higher incidence of PTSD [16, 19–21]. Further, there is the incidence of enhanced PTSD symptoms if the patients have an existing chronic illness/non-communicable disease [18, 22, 23].

Other factors contributing directly to PTSD-like symptoms are intensive care units (ICU) admissions and non-invasive ventilation [12, 22]. Psychological distress at the onset of illness, pre-existing psychological disease, and psychotropic drugs are predictive or elevated distress in patients [12, 14, 18–20, 24]. Another directly contributing factor to COVID-19 related PTSD is negatively self-rating one's wellbeing status and high perceived risk of COVID-19 [14, 16, 19, 24]. When patients are concerned about infecting others, especially their loved ones, or are being discriminated against, it leads to a higher possibility of distress [24]. COVID-19 symptom load and hospitalization define PTSD symptom severity [12, 20, 25].

A belief that adequate information is provided to patients related to COVID-19 decreases PTSD symptom severity [11]. Economic stability is protective for excessive distress pertaining to COVID-19, and so is retirement [11, 16, 18]. Socially supportive network tiers while maintaining social distance, living with families, and feeling connected contribute to decreased incidence of COVID-19-related PTSD [13, 14, 16, 25]. A study from China concluded that C-reactive protein (CRP) positively correlated to increased PTSD symptoms in patients [21]. Reports of elevated post-traumatic stress symptoms related to continuous traumatic stress in contrast to patients exposed to trauma that has ended were published in a study from Israel [16]. Another study from Israel documented that higher post-traumatic growth scores attributed to prior trauma were associated with higher COVID-19-related PTSD symptoms [19]. A study from New York, USA, found that the patients who had chronic PTSD had significantly decreased PTSD symptoms relative to trauma-exposed healthy individuals during the COVID-19 pandemic [17].

The current situation in India is alarming, where COVID-19 infection has become so rampant that hospital beds are not available, nor is oxygen. Morgues and crematoriums are struggling with an increasingly large number of dead bodies. According to the latest report by World Health Organization (WHO, 12 May 2021), the total confirmed cases have risen to 2, 29, and 92,517, while total deaths are 249,992 [26]. Subhashish Mohanty et al. 2021 has reported a case of a 65-year-old man who committed suicide after getting positive COVID-19 result on 5 May 2021. After his health deteriorated, his family members put him in social isolation; suffering from mental trauma, he hung himself from a tree [27]. Reports like these clearly show that people, especially the elderly, suffer tremendously at the hands of COVID-19 infection spread. Individuals are not even turning up for cremation and burial of their relatives. Similarly, in Nepal, the rates of positive tests have risen to 44%, more than half of what it was a month ago, while only 1% of the population is vaccinated [28]. With an increase in rate and severity of COVID-19 infection, the number of hospitalized patients and mourning population is on the rise as well. This, combined with less advanced healthcare systems, can increase the social isolation and loneliness, thus prompting greater psychopathology after remission [29].

Rehman et al. (2021) has discussed how the current pandemic has affected Indian people in various ways depending on their socio-economic status and profession [30]. For instance, a frontline healthcare worker is more fearful than those working from their home, such as software engineers, and a person who is economically unstable and has to worry about the daily need of his/her family is more distressed than the person who is stable [30]. Since the whole world is suffering due to the pandemic, irrespective of being exposed to the virus, people might feel anxious regarding contracting the disease, falling sick, or even dying, leading to feelings of helplessness or even mental breakdown [31]. With a large number of confirmed cases and death from the corona virus and its socio-economic consequences worldwide, this has been the most traumatic event compared to all the disease outbreaks in recent times [32]. Uncertainties related to this novel virus, failure of the national healthcare system to handle the crisis effectively, and lack of health care infrastructure set ripple effect and worsen paranoia and anxiety during this pandemic [32]. Studies have shown that subjective experience of fear is associated with PTSD development more than the objective experience [33]. Negative or sensational media reports and witnessing the severity of disease in people all around the globe can stimulate the subconscious perception of threat and induce a subjective experience of fear in people [33].

People with PTSD are at amplified risk of suicidal ideation, attempts, and even death by suicide compared to the general population; however, they are less likely to seek help because of the fear of stigmatization, belief that symptoms will decrease gradually, and sometimes lack of enough information about the disease itself [34]. Measures should be taken to prevent people from developing PTSD in the first place and provide them care and treatment after they develop symptoms of PTSD. The Centers for Medicare & Medicaid Services (CMS) recommend the nursing home residents be involved in safe communal activities by maintaining social distancing during the lockdown time to combat isolation [35]. Book clubs, bingo, movies, therapy animals, physical contact with the loved ones through plastic protective barriers are few examples of activities that can help people deal with these stressful times [35]. Experts have suggested the use of telehealth for the early identification and treatment of PTSD and other mental health disorders in those who are experiencing significant stressors [35]. Society and country also must provide its people with help regarding jobs, medical care, and basic needs such as food, housing, education, which would ultimately help reduce the distress during this time of pandemic [35]. Conventional mental health improvement programs such as self-help groups, 12-step programs, and spiritual groups, have already started using online-based platforms to continue helping people [35]. A meta-analysis performed by Olthuis et al. in 2016 concluded that distant-delivered cognitive-behavioral therapy (CBT) successfully reduced PTSD [36]. Two other meta-analyses (Lewis et al. 2019; Sijbrandij et al. 2016) on CBT provided through the internet to PTSD patients also yielded similar results [36]. An uncontrolled open trial performed by Spence et al. in 2013 found that combined internet-delivered CBT and online Eye Movement Desensitization and Reprocessing (EMDR) reduced the severity of PTSD in adults and children [36]. There is a need to perform further studies to study the effects of web-based EMDR on PTSD, though [36]. Nonetheless, these internet-delivered therapies could be vital in managing PTSD among COVID-survivors in these COVID-inflicted times when in-person therapies are risky.

The underlying mechanisms for the risk of psychiatric ailments in people infected with COVID-19 are still unclear. Some of the reasons can be the negative psychological effects, the fear of quarantine and social isolation, uncertainty and death, compromised health after severe viral infections, economic burden, and even misinformation. One study even found the prevalence of post-COVID PTSD similar to that of disaster situations (5–10%) [12]. Also, PTSD prevalence is related to the severity of the traumatic events [37]; we can assume that COVID-19 infection is more likely to be traumatic for patients who develop severe symptoms.

An existing psychiatric illness was also a significant risk factor for the development of COVID-19-associated PTSD. A study found that out of 33.5% of patients with a pre-existing psychiatric condition, 6.5% developed PTSD at 4 weeks follow-up [12]. These results suggest that, as classically described in the PTSD literature, COVID-19-related PTSD can arise from the association between patients’ psychological vulnerability and the traumatic impact of the disease, which is also the reason why there is a high prevalence of COVID-19-associated PTSD during the acute phase [38].

It is still unknown how long the survivors can have persistent PTSD symptoms, making it difficult for psychiatrists to apply classical PTSD treatment strategies. Few studies found that symptoms of PTSD could also appear months later [39]. Others found that the prevalence of PTSD among SARS survivors was 9.8% in the early recovery phase and increased up to 25.6% after 30 months [40]. It is too early to consider that the present results reflect the definitive impact of the epidemic, and future studies should investigate the PTSD prevalence among COVID-19 patients more than 1 month after this traumatic experience. In addition, the mean age to develop psychiatric disorders and suicide in patients during the Severe Adult Respiratory Syndrome (SARS) outbreak was 3.42 (SD = 2.80) years [41]. Therefore, it is crucial for the physicians taking care for the COVID-19 survivors to monitor their mental health conditions in the first 3–4 years, especially for PTSD/acute stress disorder.

The “cytokine storm” is classically associated with a severe COVID-19 infection [42]. Patients are found to have higher levels of inflammatory cytokines like interleukin (IL)-1β, IL-4, IL-6, IL-10, interferon (IFN)-γ, CXCL10, and CCL2 [43]. Several psychiatric illnesses, including depression, bipolar mood disorder, obsessive-compulsive disorder, etc., are associated with high cytokine levels and associated inflammatory state [44, 45]. Thus, further research is needed on how these inflammatory biomarkers are associated with various psychiatric illnesses, including PTSD in COVID-19 survivors. This way, it may be possible to identify new specific targets to treat inflammation-related neuropsychiatric conditions [46].

Elderly people are the most affected by COVID-19 infection in terms of mortality, reaching 14.8% for individuals over 80 versus 0.2% for individuals under 40 years [47]. They are prone to COVID-19 infection more than the general population. They are also less able to follow the CDC-recommended preventive guidelines like wearing masks, hand hygiene, or social distancing/isolation because of cognitive issues. Loneliness and psychiatric disorders are more prevalent in this age group, further enhanced because of the COVID-19 pandemic. Moreover, they are less accustomed to the use of mobile phones or telepsychiatric services, and with restrictions to public transport due to COVID-19, it has become challenging for this population to seek professional mental health care. There is an imminent need to create more awareness among not only psychiatrists, psychologists, but also public health officials and infectious disease physicians on post-COVID PTSD in the elderly population. It is essential to effectively identify these vulnerable individuals at greater risk of developing PTSD due to the current pandemic and the associated quarantine. We need to build more and better psychological support systems, in-person or virtual, crisis interventions that can help reduce anxiety and post-traumatic stress in this population. It is important to reinforce the factors that can help in successfully containing the infection spread and greater quarantine compliance alongside developing extra support system for high-risk individuals, i.e., those at higher risk of the adverse psychological and social consequences of quarantine [48]. Governments can implement more effective methods to dismiss the unrealistic information regarding COVID-19 and the vaccination, spreading information regarding proper containment methods, and financially supporting the elderly population. These steps are essential to combat the rising morbidity and mortality of the current pandemic. Consequently, an epidemiological monitoring system is currently being set up concerning COVID-19 units in psychiatry, supporting the National Coordination of Regional Clinical Research Mechanisms in Psychiatry and Mental Health. This system will effectively calculate the efficacy of the preventive measures, morbidity, mortality, and access to recovery centers. Such a monitoring system can be dedicated to the care of all patients with psychiatric illness to record the progression of infectious morbidity/mortality, monitor treatments, and the number of psychiatric decompensation episodes/complications [49].

Utilizing positive coping mechanisms like physical and mental exercises, social support utilization, being in touch with family and friends, and diverting thoughts from the current pandemic, have seen to be beneficial to deal with the stress of COVID-19 [18]. These findings in a Georgian sample agree with previous studies to reduce psychological stress [50]. It is important to promote cognitive and behavioral coping strategies at the national level as simple primary prevention advice. Similarly, tobacco and alcohol consumption should also be strongly discouraged.

Peritraumatic distress is a crucial forecaster of post-traumatic stress disorder, but even this is not specific to COVID-19. China developed a COVID-19 Peritraumatic Distress Index (CPDI), which is a rapid data-gathering tool (10 min). The psychometric properties of the Italian version are satisfactory and confirm that CPDI is a tool that is fast, non-intrusive, administered online, and therefore “safe” in the acute settings. It allows identifying person’s requirements and suggesting rapid interventions [15].

With a rise in the number of intensive care unit (ICU) survivors, there has been an increase in psychological morbidity in patients and their families. One study highlighted that ICU diaries could potentially help reduce ICU-related PTSD and anxiety if appropriate psychiatric follow-up care is available. Clinicians must be prepared to appreciate the severity and prevalence of psychiatric complications of grave diseases [51].

Total or partial closure of mental health facilities is especially problematic. Still, regular appointments via videoconferencing with the patients and arranging home visits and specific case-management can be organized to support these individuals after hospital discharge, helping them cope with confinement, which can induce a recurrence of mental disorders. It can also monitor high-risk suicidal patients, detect and treat early warning signs of PTSD, and provide psychoeducation. Psychiatrists and clinicians can also implement measures to improve the sleep quality of these individuals, providing them with stress management and coping strategies [49].

## Conclusion

Amidst the COVID-19 pandemic, we should reorganize our health infrastructure and create more emergency health services, and be prepared to manage the confinement-related negative mental health issues that may linger even after the pandemic is over. It is only logical that we should be well equipped for such epidemics with particular attention to mental health care. More than ever, it is vital to expand scientific knowledge to optimize clinical, therapeutic, and organizational decision-making and draw lessons for future epidemics.

## Data Availability

Data sharing is not applicable to this article as no datasets were generated or analyzed during the current study.

## Abbreviations

COVID-19: Coronavirus disease-19
PTSD: Post-traumatic stress disorder
CDC: Centers for Disease Control and Prevention
ICU: Intensive care unit
CRP: C-reactive protein
WHO: World Health Organization
CMS: Centers for Medicare & Medicaid Services
CBT: Cognitive behavioral therapy
EDMR: Eye Movement Desensitization and Reprocessing
SARS: Severe Adult Respiratory Syndrome
IL: Interleukin
IL-1β: Interleukin 1 beta
IFN-γ: Interferon-gamma
CXCL10: C-X-C motif chemokine ligand 10
CCL2: C-C motif chemokine ligand 2
CPDI: COVID-19 Peritraumatic Distress Index
